# Bioconversion of glycerol to ethanol by a mutant *Enterobacter aerogenes*

**DOI:** 10.1186/2191-0855-2-20

**Published:** 2012-03-29

**Authors:** RES Nwachukwu, A Shahbazi, L Wang, S Ibrahim, M Worku, K Schimmel

**Affiliations:** 1 Energy and Environmental Systems, North Carolina A & T State University, Greensboro, NC, USA; 2 Biological Engineering Program, North Carolina A & T State University, Greensboro, NC, USA; 3 Food and Nutritional Sciences, North Carolina A & T State University, Greensboro, NC, USA; 4 Department of Animal Sciences, North Carolina A & T State University, Greensboro, NC, USA; 5 Biological Engineering Program, Sockwell Hall, North Carolina A & T State University, 1601 East Market Street, Greensboro, NC 27411, USA

**Keywords:** *Enterobacter aerogenes*, Ethanol, Glycerol, Biodiesel, Fermentation

## Abstract

The main objective of this research is to develop, by adaptive evolution, mutant strains of *Enterobacter aerogenes *ATCC 13048 that are capable of withstanding high glycerol concentration as well as resisting ethanol-inhibition. The mutant will be used for high ethanol fermentation from glycerol feedstock. Ethanol production from pure (P-) and recovered (R-) glycerol using the stock was evaluated. A six-tube-subculture-generations method was used for developing the mutant. This involved subculturing the organism six consecutive times in tubes containing the same glycerol and ethanol concentrations at the same culture conditions. Then, the glycerol and/or ethanol concentration was increased and the six subculture generations were repeated. A strain capable of growing in 200 g/L glycerol and 30 g/L ethanol was obtained. The ability of this mutant, vis-à-vis the original strain, in utilizing glycerol in a high glycerol containing medium, with the concomitant ethanol yield, was assessed. Tryptic soy broth without dextrose (TSB) was used as the fermentation medium. Fermentation products were analyzed using HPLC.

In a 20 g/L glycerol TSB, *E. aerogenes *ATCC 13048 converted 18.5 g/L P-glycerol and 17.8 g/L R-glycerol into 12 and 12.8 g/L ethanol, respectively. In a 50 g/L P-glycerol TSB, it utilized only 15.6 g/L glycerol; but the new strain used up 39 g/L, yielding 20 g/L ethanol after 120 h, an equivalence of 1.02 mol ethanol/mol-glycerol. This is the highest ethanol yield reported from glycerol bioconversion. The result of this P-glycerol fermentation can be duplicated using the R-glycerol from biodiesel production.

## Introduction

There is an increasing demand for biofuels alternatives to petroleum-based fuel due to the health and environmental problems of the latter. Moreover, fossil fuel is not renewable; Campbell and Laherrere (1998) predict that petroleum reserves will be completely depleted by 2050. Recently, there has been a significant increase in the production and use of bioethanol and biodiesel. These biofuels - apart from being alternatives to fossil-derived fuels - are secure, renewable, non toxic, have a favorable energy balance and lower harmful emissions and are, therefore, environmentally friendly. Biodiesel is produced from the transesterification of vegetable oils or animal fats using simple alcohols (methanol or ethanol) and alkali catalysts. The process generates a lot of glycerol as a by-product. Specifically, the amount of glycerol generated is about 10% of the biodiesel produced (Yazdani and Gonzalez, 2008). Thus, for every 100 lb of biodiesel produced, 10 lb of glycerol is generated as waste. It is rightly predicted that crude glycerol availability will increase in the near future due to this global growth in biodiesel production (Dharmadi *et al.*, 2006).

Crude glycerol from biodiesel production presents great economic and environmental challenges. It is expensive to purify, and its improper disposal can contaminate the lithospheric environment. Yet, its surplus collapses the price of glycerol, which affects the glycerol-producing and -refining industries. Consequently, the economic viability of biodiesel industry hangs on the balance, unless the market value of the glycerol by-product is improved. In fact, some of these industries are threatened with bankruptcy (Wilke and Vorlop 2004). Currently, glycerol-producing plants owned by some chemical companies, such as Dow chemical, and Procter and Gamble Chemicals, have been shut down (McCoy, 2006). Therefore, development of processes to convert this low-value glycerol to higher value products is an excellent opportunity to improve the economic viability of biodiesel production, and also make it environmentally safer.

Chemical and biological approaches to the conversion of biodiesel waste into high value products are currently being explored. Chemical catalysis has many disadvantages. They include: low product specificity, need for high pressure and/or temperature, and inability to use crude glycerol with high levels of contaminants (Yazdani and Gonzalez, 2007). But biological approaches through either aerobic or anaerobic fermentation hold better promise. Anaerobic fermentation is preferred over aerobic because the capital and operational costs involved in the former are less than in the later (Yazdani and Gonzalez, 2007):

i) Anaerobic fermenters are less expensive to build and operate than aerobic ones;

ii) Anaerobic fermenters use less energy than aerobic counterparts.

It is proposed that biofuels industries should also establish biorefinaries which convert co-products to higher value products to achieve increased economic viability (Kamm and Kamm, 2007; Yazdani and Gonzalez, 2007). A biofuel industry which has biorefinaries that convert waste into higher value products could achieve this purpose. The glycerol-rich streams of waste generated during biodiesel production have the potential to be used in the proposed technology.

Glycerol is a good carbon and energy source for many microorganisms, and, therefore, can be an invaluable feedstock for industrial fermentations (Da Silva et al., 2009). The ability to ferment glycerol in the absence of air is found in a few members of *Enterobacteriaceae *(Bouvet *et al.*, 1995). Fewer members of this family of bacteria namely *Citrobacter, Klebsiella, Enterobacter*, and *Escherichia *have been reported to produce ethanol as a major product of anaerobic fermentation of glycerol (Gonzalez *et al.*, 2008; Homann *et al.*, 1990; Ito *et al.*, 2005; Jarvis *et al.*, 1997; Lin, 1976; Streekstra et al. 1987). Other co-products include hydrogen, 1, 3-propanediol, succinate, lactate, acetate, propionate, formate, and 2, 3-butanediol. However, the ethanol production was very slow and the quantity too low in all but one of the reported cases. *Enterobacter aerogenes *is the only species reported to produce ethanol, hydrogen and carbon dioxide as the main products (Ito *et al.*, 2005). It is a facultative anaerobe and can be utilized for high-yield production of ethanol from crude glycerol. The biological fermentation of glycerol into ethanol and H_2 _is attractive because H_2 _is expected to be a future clean energy source while ethanol can be used as raw material, a supplement to gasoline, and a feedstock for biodiesel production in place of methanol (Sakai and Yagishita, 2007).

The maximum theoretical yield of ethanol and hydrogen (or formate) from glycerol dissimilation is 1 mol each of ethanol and hydrogen (or formic acid) per mol of glycerol utilized.

C_3_H_8_O_3 _-----------> C_2_H_5_OH + H_2 _+ CO_2_

 Or

C_3_H_8_O_3 _ -----------> C_2_H_5_OH + HCOOH

This means that 50% glycerol is theoretically converted to ethanol, 2.2% is converted to H_2_, and 47.8% is converted to CO_2_; or 50% glycerol is converted to ethanol, and 50% is converted to formic acid.

This paper presents the result of research using *E. aerogenes *ATCC 13048 to ferment pure (P-) and recovered (R-) glycerol into ethanol. It also discusses the development of a mutant strain of the named bacterium, which is capable of growing in a high glycerol concentration and of resisting product (ethanol) inhibition. Finally, this paper reports the result of using the new strain to convert P-glycerol to ethanol vis-à-vis the original strain.

## Materials and methods

### Recovery of glycerol from biodiesel waste

To 1000 ml of crude glycerol in a 2.5 L beaker was added 60 ml of 85% H_3_PO_4_. The mixture was stirred with a magnetic stirrer, dispensed into four 250 ml serum bottles and centrifuged at 3400 rpm and 25°C for 20 min using a Centra-GP8R centrifuge (Thermo IEC, Needham Heights, MA, USA), rotor 216-A. The precipitated salt settled at the bottom. The supernatant was poured into a separatory funnel and was allowed to phase separate for 5-10 min. The free fatty acids (FFA) formed the upper layer, while the glycerol was recovered from the bottom. The recovered glycerol(R-glycerol) was analyzed with a Shodex RSpak KC-811 column HPLC (Waters Corporation, Milford, MA) to determine the percentage composition of glycerol. The precipitated salt was analyzed using Ion Coupled Plasma (ICP) Spectroscopy.

### The inoculum

*E. aerogenes *ATCC 13048 was cultivated using sterilized regular tryptic soy broth (n-TSB) and agar (n-TSA). Sterility was achieved by autoclaving the media for 15 min at 121°C. The organism was first grown for 24 h at 37°C in n-TSB from where it was cultivated on n-TSA plates by streaking to establish purity. A pure colony on the plate was subcultured on fresh sterile n-TSB for 24 h at 37°C, kept at 4°C, and labelled "stock". The stock was used for mutant development and to prepare inocula for the fermentations. The inoculum was prepared by aseptically inoculating the organism into a fresh, sterile n-TSB and incubating it for 18-24 h at 37°C. It was then washed thrice with 0.1% peptone water and re-suspended in sterile TSB with the same glycerol concentration as the fermentation medium. It was then used to aseptically inoculate the fermentation broth such that the inoculum made up 4% of the broth. This ensured that the glycerol concentration of the fermentation broth was not reduced due to the inoculum.

### Mutant development

We labeled the stock broth culture (wild strain) S001 and the agar culture plate, P1. This P1 was used to progressively develop mutant strains that withstood 50, 100, 150, and 200 g/L glycerol concentrations. We used a six-tube-subculture-generations (6 TSG) technique to achieve this. This technique, based on the principle of adaptive evolution, involved subculturing the organism six consecutive times in tubes of TSB containing the same glycerol at the same concentration and culturing conditions. Then, the glycerol concentration was increased and the six subculture generations repeated. We obtained strains that grew in 200 g/L glycerol and labeled the tube S005. Starting from S005, the same technique was employed to develop another strain that grew in a medium containing 20% (w/v) glycerol and 3% (v/v) ethanol (200 G +30E medium). This was labeled S012. The three strains, namely S001, S005 and S012, were preserved in 15% glycerol at -80°C. Their agar plates (P1, P5, and P12) were kept at 4°C, and were subcultured every 4 weeks.

### Fermentation of P- and R-glycerol

We used tryptic soy broth without dextrose (TSB) as base for the fermentation medium. Various concentrations of P-glycerol namely 5, 10, 15, and 20 g/L, were added separately to the fermentation media, mixed with magnetic stirrer bar, and 48 ml dispensed into 125 ml serum bottles. Each concentration was prepared in triplicates. We created anoxia by purging the headspaces of the bottles with nitrogen gas for two minutes. Each bottle was sealed with black butyl rubber stopper, autoclaved at 121°C for 18 min, and inoculated by using hypodermic syringe to inject 2 ml of the inoculum. The needle was not removed but had a 0.45 μm filter fitted to its base. This prevented air from entering, but allowed gases produced to escape, preventing pressure build-up that could interfere with the bacterial growth and function. Then the bottles were incubated at 37°C and 120 rpm for 48 h. We took fermentation samples at twenty-four-hour intervals beginning from 0 h, and analyzed them using HPLC (Waters Corporation, Milford, MA) with a Shodex RSpak KC-811 column and 0.1% H_3_PO_4 _as the mobile phase (1.0 mL/min, 60°C).

The fermentation was repeated by replacing P-glycerol with R-glycerol. The results were recorded. Duplicates of two sets of controls were also prepared and incubated like the rest. The first set contained 50 ml of the fermentation broth with glycerol, but no organism was inoculated. This would help to determine whether the products were actually fermentation products of the organism. The other set of controls contained the fermentation base medium and the inoculum, but no glycerol. This would help to determine whether the products actually came from the glycerol. Finally, we used the new strain as inoculum for two sets of P-glycerol fermentations, which were incubated for 48-120 h. The fermentation broth in the two sets contained 25 and 50 g/L glycerol, respectively. A third set had 50 g/L P-glycerol and the stock inoculum, which helped to compare the activity of the stock against the new strain.

The wild strain (S001) and the mutant (S012) were grown and incubated for 9 h at 37°C in three different media compositions to compare their growths. The media include TSB, TSB + 0.5% dextrose, and TSB + 20% P-glycerol.

## Results

### Recovered glycerol

The HPLC analysis of the recovered glycerol showed that it contained 69.8% glycerol, 27.4% methanol, and trace amounts of free fatty acid; the ICP spectroscopy analysis showed that the precipitate was a potassium salt. The slightly turbid appearance of the recovered glycerol indicates the presence of the precipitated potassium salt.

### Fermentation of pure glycerol

**Figure 1:**
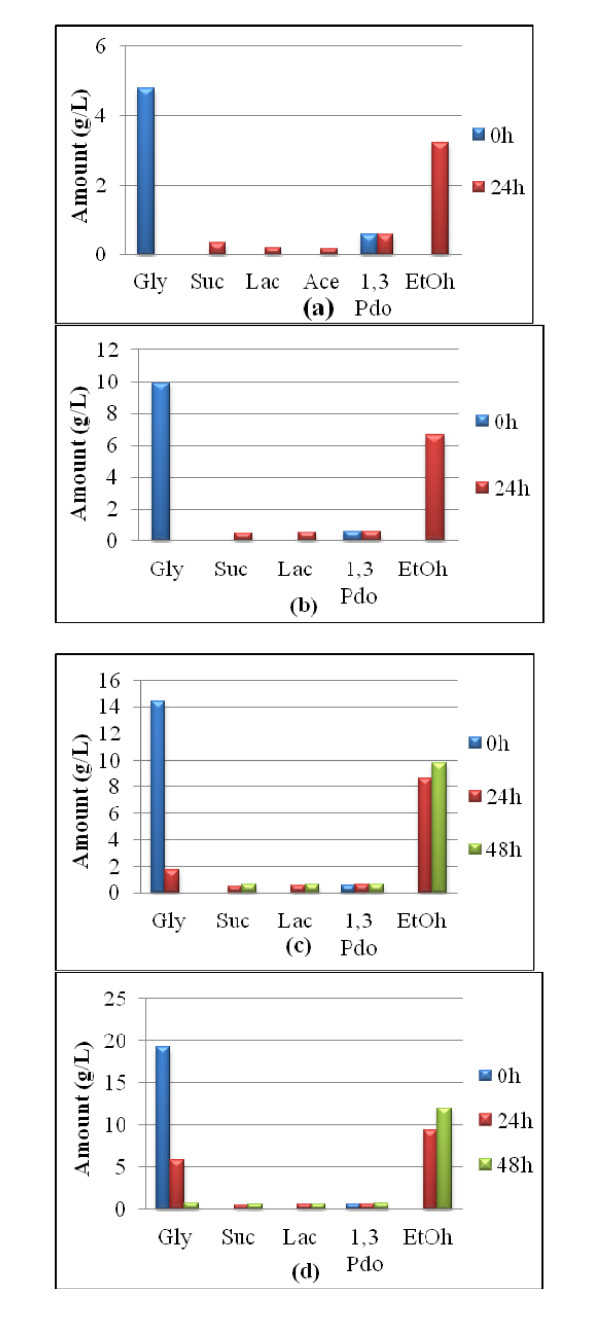
**P-Glycerol Fermentation using *Enterobacter aerogenes *ATCC 13048**. **(a) **0.5% P-glycerol **(b) **1.0% P-glycerol **(c) **1.5% P-glycerol **(d) **2.0% P-glycerol.

**Figure 2:**
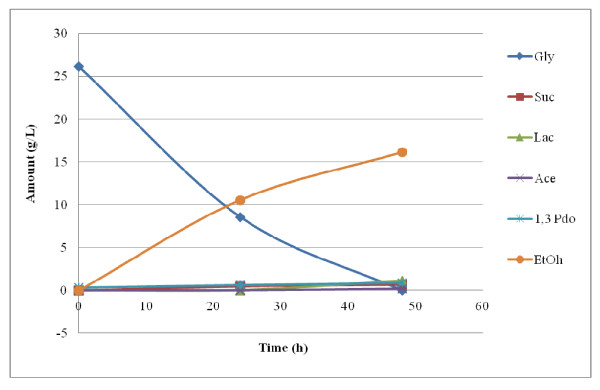
**2.5% P-Glycerol Fermentation using *Enterobacter aerogenes *S012**.

**Figure 3:**
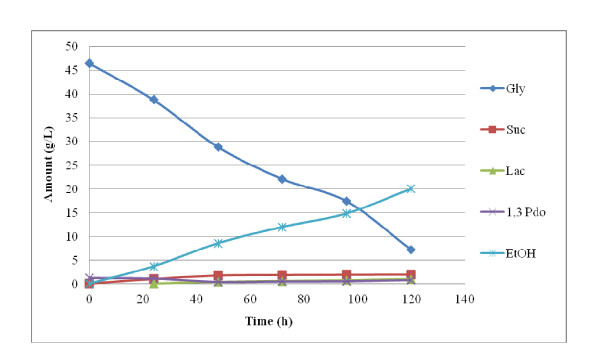
**5% P-Glycerol Fermentation using *Enterobacter aerogenes *S012**.

The result of the fermentation of P-glycerol is summarized in Figures 1, 2, 3. Figure 1 shows that *E. aerogenes *ATCC 13048 was at its best for utilizing glycerol within 48 h. It yielded about 12 g/L ethanol from 18.5 g/L of the feedstock within 48 h at a glycerol concentration of about 2% (w/v). This means that about 70% of the used glycerol was converted to ethanol. This is equivalent to 1.29 mol ethanol/mol glycerol. However, when the feedstock concentration was 50 g/L, its utilization dipped: only 15.6 g/L was utilized even after 96 h (data not shown). Moreover, acetate production was only observed at 5 g/L glycerol concentration. Figure 2 shows that *E. aerogenes *S012, the new strain, used up 26 g/L glycerol within 48 h, yielding 16 g/L ethanol. This is 1.23 mol ethanol/mol glycerol. This demonstrated that the new strain utilized glycerol with the concomitant ethanol yield more than the stock strain. As observed in Figure 2, acetate and lactate were found only at 48 h. Again, Figure 3 shows that the new strain used 39.3 g/L glycerol in 120 h to produce 20 g/L ethanol, equivalent to the theoretical maximum of 1.02 mol ethanol/mol glycerol. This indicated that a very high glycerol concentration, up to 50 g/L, reduced the ability of the new strain to utilize it. It nonetheless established the fact that the new strain is better adapted to utilize glycerol and effectively convert it to ethanol than the stock.

**Figure 4:**
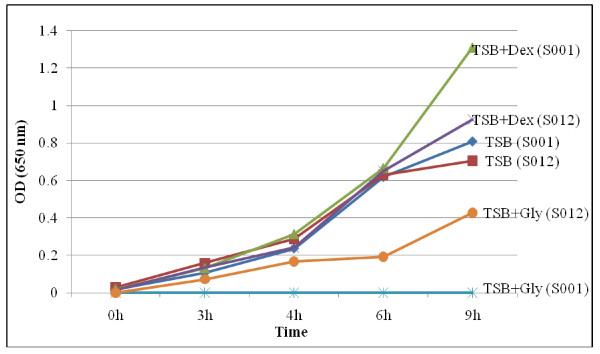
***E. aerogenes *S001 and S012 Growth Curves**. Cultural conditions: P-Glycerol, 20%; dextrose, 0.5%; Initial inoculum, ~3 log CFU/ml; pH 7.0; Temperature, 37°C. Final cell count (after 9 h): TSB (S001), 7.2 log CFU/ml; TSB (S012), 6.3 log CFU/ml; TSB + Dex (S001), 8.2 log CFU/ml; TSB + Dex (S012), 7.3 log CFU/ml; TSB + Gly (S001), < 3 log CFU/ml; TSB + Gly (S012), 4.2 log CFU/ml.

Figure 4 shows that S001, the wild strain grew better than S012, the mutant, in TSB and TSB + dextrose. It also shows that dextrose improved the growth of either strain in TSB. However, S001 was unable to grow in TSB containing 200 g/L glycerol whereas S012 did. This confirms that S012 is a mutant of S001, and that it withstands high glycerol concentration more than the wild. Although the high glycerol concentration reduced the growth of the mutant, it was observed that the growth rate more than doubled after 6 h, showing that growth improved with time.

### Fermentation of recovered glycerol

**Figure 5:**
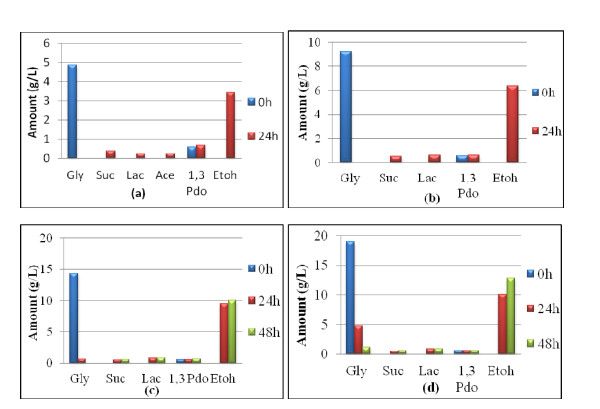
**R-Glycerol Fermentation using *Enterobacter aerogenes *ATCC 13048**. **(a) **0.5% R-glycerol **(b) **1.0% R-glycerol **(c) **1.5% R-glycerol **(d) **2.0% R-glycerol.

The result of the R-glycerol fermentation to ethanol by *E. aerogenes *ATCC 13048 is summarized in Figure 5. About 12.8 g/L ethanol was obtained from 17.8 g/L glycerol, which is more than 70% used glycerol-to-ethanol conversion. This demonstrates that the organism utilized less but converted more R-glycerol to ethanol than P-glycerol. As observed in P-glycerol fermentation, acetate was also produced at low R-glycerol concentrations, all the glycerol was not utilized within 48 h, and glycerol-to-ethanol conversion efficiency was high.

The control experiment without inoculum had no fermentation products (data not shown). Likewise the second control with the organism but no glycerol (data not shown). This established the fact that *E. aerogenes *was solely responsible for the fermentation of glycerol.

## Discussion

The results reported in Figures 1 and 3, which showed that *E. aerogenes *ATCC 13048 was at its best for utilizing glycerol within 48 h, suggest that the microorganism suffered some product inhibition. Another reason could be depletion of nutrients in the medium. In all fermentations involving 2% glycerol (pure and recovered) using either the original organism or the new strain, more than 1.0 mol-ethanol was produced per mol-glycerol. This is not the first time a glycerol bioconversion experiment has yielded a product above the theoretical maximum. In their report on hydrogen and ethanol production from glycerol, Ito *et al. *(2005) observed that *E. aerogenes *HU-101 produced 1.12 mol H_2_/mol-glycerol, which was 0.12 mol higher than the theoretical maximum. Yazdani and Gonzalez (2008) also reported an ethanol yield of 1.04 mol/mol-glycerol from *E. coli*, 0.04 mol above the theoretical maximum. The reason could be the presence of unknown carbon and/or electron sources in the medium or that amino acids contained in the complex medium served as additional carbon for the bacteria. This research observed that the higher the glycerol concentration, the more was utilized, up until about 20 g/L of feedstock concentration, agreeing with Ito *et al. *(2005) who reported that the higher the concentration of P-glycerol up to 25 g/L, the more was utilized. This work also found ethanol yield proportional to glycerol utilization. This is contrary to Ito *et al. *(2005) who reported that though the rate and amount of glycerol utilized at higher concentration up to 25 g/L increased, lower ethanol was produced.

*E. aerogenes *ATCC 13048 utilized less R-glycerol than P-glycerol, yet the efficiency at which it converted the former to ethanol was higher than for the latter. The higher conversion efficiency of R-glycerol to ethanol than P-glycerol by *E. aerogenes *ATCC 13048 is probably due to the lower pH of R-glycerol fermentation broth, shown in Table 1. (The lower pH obviously came from residual phosphoric acid used during the recovery of glycerol from the crude.) It has been reported that *E. aerogenes *grows best at neutral pH (Nakashimada et al., 2002) while it requires acidic medium for optimum product formation (Ito et al., 2005; Nakashimada et al. 2002). Ito *et al. *(2005) reported that biodiesel waste above 1.7 g/L did not do well in ethanol production due to high salinity. Similarly, Gonzalez *et al. *(2008) discovered that K^+ ^and PO_4_^3- ^were inhibitory to enteric bacteria growth and fermentation of glycerol. We found in this work that removal of salt by acidification improved R-glycerol utilization and ethanol yield by *E. aerogenes *ATCC 13048.

**Table 1:** Initial pH of the P-glycerol & R-glycerol Fermentation Broth

Gly Conc. (%)	P-Gly broth initial pH	R-Gly broth initial pH
0.5	7.15	7.01

1.0	7.16	6.92

1.5	7.17	6.84

2.0	7.18	6.78

2.5	7.2	-

The work of Yazdani and Gonzalez (2008) produced a genetically modified *E. coli *that yielded ethanol equal to the theoretical maximum from 10 g/L glycerol. That was the highest reported microbial glycerol-to-ethanol conversion. But in the current research, we obtained an organism that utilized much higher concentration of glycerol with similar, if not better, glycerol-to-ethanol conversion efficiency.

In conclusion, *Enterobacter aerogenes *promises to be a better organism for the conversion of crude glycerol - waste product of biodiesel production - into ethanol. The bacterium has demonstrated an ability to produce fewer co-products at trace concentrations as the glycerol concentration gets higher. This microbial stock effectively and efficiently converted P- and R-glycerol, at concentrations of 20 g/L or less, into over 10 g/L ethanol. The new mutant strain converted 26 g/L glycerol into 16 g/L ethanol in 48 h, but could not operate at the same efficiency when glycerol concentration reached 50 g/L. Yet the ability of the new strain to convert glycerol to ethanol at effectiveness of 1 mol ethanol/mol glycerol in high glycerol-containing medium is potentially of great value to biofuels industry. Thus, we can safely conclude that *E. aerogenes *S012 can utilize high amounts of recovered glycerol and effectively convert same to ethanol.

Further work is required to obtain the optimum cultural conditions for the utilization of glycerol and yield of ethanol by this *E. aerogenes *S012. The optimized conditions should then be applied to convert waste streams from biodiesel production into ethanol by this bacterium.

## Competing interests

The authors declare that they have no competing interests.
